# A novel nonsense mutation of *ZEB2* gene in a Chinese patient with Mowat‐Wilson syndrome

**DOI:** 10.1002/jcla.23413

**Published:** 2020-06-10

**Authors:** Yuan Hu, Qi Peng, Keze Ma, Siping Li, Chunbao Rao, Baimao Zhong, Xiaomei Lu

**Affiliations:** ^1^ Department of Pediatrics Hematology Dongguan Children's Hospital Dongguan China; ^2^ Department of Medical and Molecular Genetics Dongguan Institute of Pediatrics Dongguan China; ^3^ Medical laboratory Dongguan Children's Hospital Dongguan China; ^4^ Key Laboratory for Children's Genetics and Infectious Diseases of Dongguan City Dongguan China; ^5^ Pediatric Intensive Care Unit Dongguan Children's Hospital Dongguan China

**Keywords:** genetic, Mowat‐Wilson syndrome, novel mutation, thrombocytopenic purpura, ZEB2

## Abstract

**Background:**

Mowat‐Wilson syndrome (MWS) is a rare genetic disorder characterized by intellectual disability, distinctive facial features, and multiple anomalies caused by haploinsufficiency of the ZEB2 gene. We investigated the genetic causes of MWS in a 14‐year‐old girl who had characteristic features of MWS.

**Methods:**

Clinical data and peripheral blood DNA samples were collected from the proband. Following extraction of genomic DNA, whole‐exome sequencing was conducted to detect genetic variants. Bioinformatics analysis was carried out to predict the function of the mutant gene.

**Results:**

Mutation analysis of the proband identified a novel nonsense mutation (c.250G > T, p.E84*) within exon 3 of the ZEB2 gene. This novel alteration resulted in a termination codon at amino acid position 84, which was predicted to encode a truncated protein. This variant was not present in unrelated healthy control samples that were obtained from the exome sequence databases ExAc browser (http://exac.broadinstitute.org/) and gnomAD browser (http://gnomad.broadinstitute.org/). It is a novel variant that was determined to be a deleterious mutation according to the variant interpretation guidelines of the ACMG. The results of our study suggest that the p.E84* mutation in the *ZEB2* gene was probably the pathogenic mutation that caused MWS in the proband.

**Conclusions:**

This study reports the novel mutation in the proband will provide a basic foundation for further investigations to elucidate the ZEB2‐related mechanisms of MWS.

**Abbreviations**
ACMG: American College of Medical Genetics
MWS: Mowat‐Wilson syndrome
NGS: next‐generation sequencing
SIP1: Smad‐interacting protein 1
ZEB2: zinc finger E‐box‐binding homeobox 2

## 1 INTRODUCTION

Mowat‐Wilson syndrome (MWS, OMIM # 235 730) is a rare autosomal dominant disorder that affects approximately one in every 50 000‐70 000 individuals.^1^ MWS is characterized by distinctive facial features (widely spaced eyes, broad eyebrows with a medial flare, low‐hanging columella, prominent or pointed chin, open‐mouth expression, and uplifted earlobes with a central depression), congenital heart defects with a predilection for abnormalities of the pulmonary arteries and/or valves, Hirschsprung disease or chronic constipation, genitourinary anomalies (particularly hypospadias in males), and hypogenesis or agenesis of the corpus callosum.^1^, ^2^ It is caused by haploinsufficiency of the ZEB2 (zinc finger E‐box‐binding homeobox 2) gene on chromosome 2q22.3 through premature stop codons or large deletions.^3^, ^4^

ZEB2, also known as SIP1 (Smad‐interacting protein 1) and ZFHX1B, is a member of the family of two‐handed zinc finger/homeodomain transcription factors.^5^, ^6^ A common structural feature of these proteins is the presence of a homeodomain separated by two clusters of “zinc finger” domains with DNA‐binding activity.^7^ It has 10 exons (exon 1 is noncoding) and is approximately 165 kb in size, with a full length of 1214 aa in human.^6^ ZEB2 acts mostly as a transcriptional repressor but can also activate the transcription of target genes together with its co‐factors.^8^, ^9^ MWS patients carry de novo heterozygous ZEB2 mutations, and most of these generate premature stop codons (85%) or deletions encompassing the gene (15%).^10^ Various mutations in MWS patients cause either complete ablation of the ZEB2 protein or the production of a nonfunctional ZEB2 protein.

In the present study, we report a 14‐year‐old girl with MWS caused by a novel ZEB2 heterozygous variant (c.250G > T, p.E84*), who had characteristic features of MWS combined with thrombocytopenic purpura on the skin all over the body. Our study will provide a basic foundation for further investigations to elucidate the mechanisms of MWS.

## 2 MATERIALS AND METHODS

### 2.1 Ethical compliance

The study was approved by the Ethics Committee of Dongguan Children's Hospital in agreement with the Declaration of Helsinki. Written informed consent was obtained from the guardians of the study subject.

### 2.2 Case presentation

A 14‐year‐old girl was admitted to our hospital because of petechiae and ecchymosis present on skin all over the body. She was an orphan who lived in a welfare institution with unknown birth status. At 3 years old, she could stand but not walk alone, and her speech was limited to a few words. Additionally, she was found to have intellectual disability and congenital heart disease (ventricular septal defect).

Approximately 1 year ago, the nurse in the welfare institution found some petechiae on her skin and sent her to a local hospital. The routine blood test results showed that the platelet count was 10 × 10^9^/L (normal reference range: 100‐300 × 10^9^/L) and progressively decreased, and then, she was diagnosed as thrombocytopenic purpura. The routine bone marrow examination showed granulocytic hyperplasia was active, and no megakaryocytes were observed. The cell morphology in the bone marrow and peripheral blood was normal.

**Figure 1:**
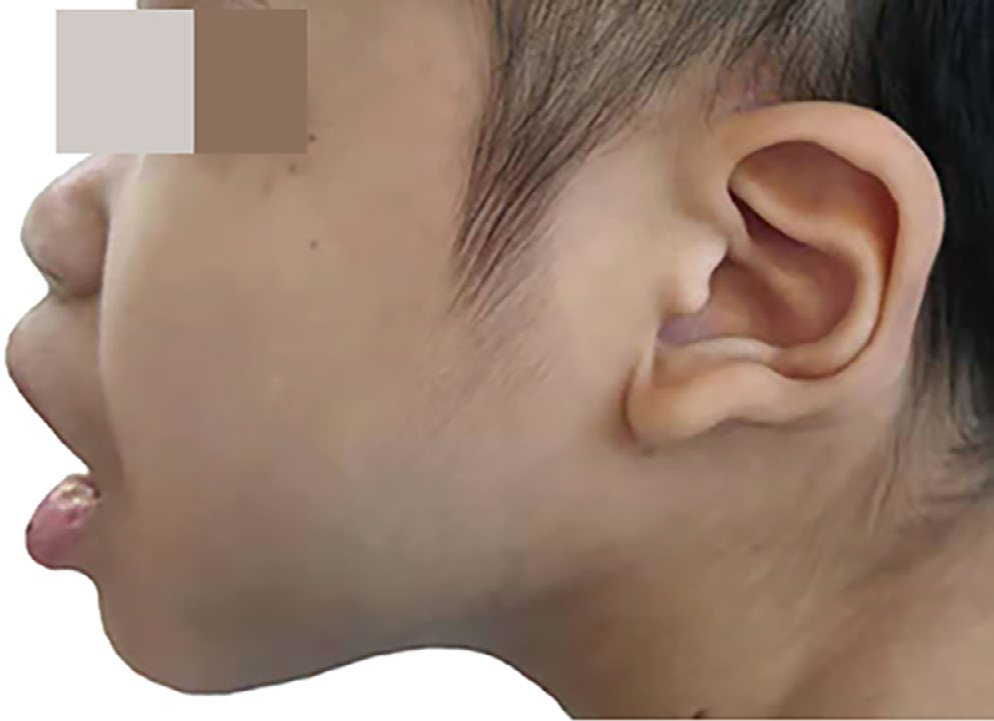
Characteristic facial appearance of our patient with Mowat‐Wilson syndrome. Microcephaly with narrow chin, thick eyebrows, widely set eyes with strabismus, cupped ears with a central depression, and uplifted lobes, open mouth, a shortened philtrum, and a wide nasal bridge

When she first came to our hospital, her distinctive physical features attracted our attention. Her height was 134 cm (<3rd centile), her weight was 23 kg (<5th centile), her head circumference was 45.5 cm (<3rd centile), and her BMI was 12.8. She had microcephaly with a narrow chin, broad eyebrows, widely set eyes with strabismus, cupped ears with a central depression, and uplifted lobes, an open mouth, a shortened philtrum, and a wide nasal bridge (Figure 1). The major joints had a normal range of motion, but she displayed increased reflexes and tone of both upper and lower body segments as well as poor coordination with a wide‐based gait. The platelets and granulocytes progressively decreased and carried a risk of infection. We tried therapy of platelet transfusion and immunoglobulin infusion, but no effects were observed. Finally, intravenous cyclosporine has proven to be an effective therapy for the symptom of thrombocytopenic purpura, and the platelet counts returned to the normal level above 100 × 10^9^/L. The symptom of epilepsy first occurred at 14 years old.

### 2.3 Genetic study

Genomic DNA was extracted from peripheral blood samples of the subject using the Blood DNA Kit (Tiangen Biotech) following the manufacturer's protocol. The DNA yield and quality were assessed using a NanoDrop 8000 UV spectrophotometer (Thermo Fisher Scientific).

Next‐generation sequencing (NGS) of the whole exome of the subject was conducted as follows: The genomic DNA was fragmented by a Q800R Sonicator (Qsonica) to generate 300‐ to 500‐bp fragments. Enriched DNA samples were indexed and sequenced with a NextSeq 500 sequencer using xGen^®^ Exome Research Panel (Integrated DNA Technologies, Inc.) and a configuration with a read length of 2 × 150 base pairs (bp). Sequencing reads were mapped to the reference human genome version hg19 (2009‐02 release, http://genome.ucsc.edu/). Nucleotide changes observed in the aligned reads were called and reviewed using NextGENe software (SoftGenetics). Sequence variants were annotated using population and literature databases, including the 1000 Genomes Project, dbSNP, GnomAD, ClinVar, HGMD, and OMIM. Possible pathogenicity was predicted according to the online tools MutationTaster, FATHMM‐MKL, and LRT. Variants were classified according to guidelines published by the American College of Medical Genetics and Genomics (ACMG).^11^

## 3 RESULTS

**Figure 2:**
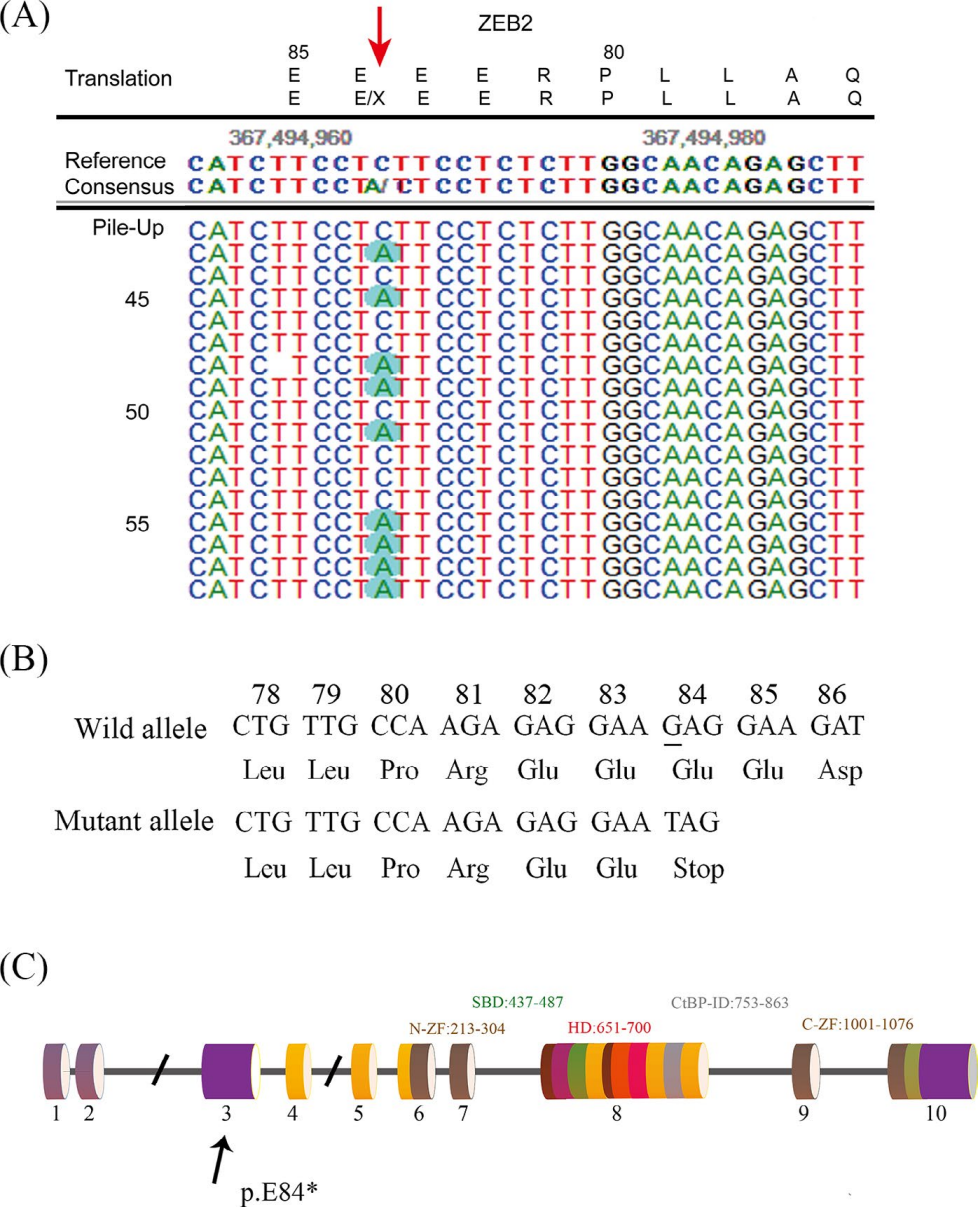
Genetic analysis of the heterozygous variant. A, DNA sequencing results of the proband. B, Note the novel mutation resulting in a premature stop codon at position 84 of the ZEB2 gene. C, Schematic representation of the ZEB2 protein and its domains, indicating the location of the novel mutation that results in premature protein termination. CtBP‐ID, C‐terminal‐binding protein‐interacting domain; C‐ZF, C‐terminal zinc finger cluster; HD, homeodomain; N‐ZF, N‐terminal zinc finger cluster; SBD, Smad‐binding domain

A heterozygous variant (c.250G > T, p.E84*) of the ZEB2 gene (GenBank reference sequence: NM_014795.4) was identified in this patient (Figure 2). It was a nonsense mutation that led to a truncated protein with termination at amino acid 84 (Figure 2). This variant was not present in unrelated healthy control samples that were obtained from the exome sequence databases ExAc browser (http://exac.broadinstitute.org/) and gnomAD browser (http://gnomad.broadinstitute.org/) and was predicted to be “disease causing” by MutationTaster, “Damaging” by FATHMM‐MKL, and “Deleterious” by LRT. Based on the ACMG criteria, there was some evidence for the pathogenicity of the novel heterozygous mutation, including one very strong (PVS1), one moderate (PM2), and one supporting (PP3) piece of evidence. Therefore, the novel mutation identified in this patient was determined to be a pathogenic mutation, which strongly suggests that this novel mutation was closely associated with MWS in this proband. No other pathogenic mutations were found in other genes in patient that are known to be associated with MWS or blood disorders.

## 4 DISCUSSION

The novel nonsense mutation c.250G > T found in exon 3 of the ZEB2 gene in this study has not been previously reported. *ZEB2* genetic variants associated with MWS have been reported in all 8 exons of the ZEB2 gene, and the majority of these variants occur in exons 3 and 8.^12^, ^13^ The majority of genetic defects involve nonsense mutations and insertion or deletions (indels) that induce a frameshift and transcription of a premature stop codon; approximately 15% of MWS patients have deletions; and large‐scale ZEB2 rearrangements account for approximately 1% of cases.^4^, ^14^, ^15^, ^16^

Almost all cases of MWS have arisen from de novo *ZEB2* mutations, but somatic and presumed parental germline mosaicism has been reported.^17^ Unfortunately, the paternal DNA of our patient was unavailable for testing. ZEB2 regulates target gene transcription through the interaction between two zinc fingers in each of its two zinc finger clusters and the CACCT(G) or CACANNT(G) sites in regulatory regions of its target genes.^18^ The novel mutation we identified leads to a truncated protein with termination at amino acid 84, and both of the zinc finger domains were absent in the mutant protein. Thus, the function of regulating target gene transcription was missing in the mutant protein.

The approximate frequencies of clinical features in historical cases and our patient's clinical findings are summarized in Table 1.^1^, ^4^, ^17^, ^18^, ^19^, ^20^ The proband in this study has characteristic features, but the symptoms of the hematological system of thrombocytopenic purpura were never reported in previous MWS cases. The symptom of purpura is rarely reported in the literature of MWS; only one case of a 10‐month‐old girl with MWS presenting with purpura fulminans was reported.^21^ It is speculated that this was because of immunodeficiency caused by asplenia in the proband, which was not different from our patient.^21^ Although the symptom of thrombocytopenic purpura has never been reported to be associated with MWS, ZEB2 has been shown to play a role in embryonic hematopoiesis and is a master regulator of multicellular differentiation in adult hematopoiesis.^22^ Previous research reports that ZEB2 is abundant in hematopoietic cells, with high levels of mRNA in HSCs and HPCs.^22^ Drug‐inducible conditional‐*Zeb2*‐inactivation mice exhibited significantly reduced leukocytes, erythrocytes, and platelets.^23^ Additionally, Zeb2, as a transcription factor, has been shown to be a novel AML dependency and is related to AML proliferation and prognosis.^24^ Infusion of platelets and immunoglobulin is ineffective, and treatment with cyclosporine A is effective, suggesting that thrombocytopenia in the proband may be caused by autoimmunity. However, the association between the novel ZEB2 mutation and the symptom of thrombocytopenic purpura in the proband needs to be further studied.

**Table 1:** Clinical Features in Mowat‐Wilson Syndrome by Frequency

Clinical features	Approximate frequency（%）	Present patient
Facial dysmorphism		
Round or square face ininfancy, longer face in childhood		
Hypertelorism, deep‐set but large eyes		
Broad nasal bridge		
Medially flared and broad eyebrows, heavier and broad eyebrows in adolescence	100	+
Prominent columella		
Prominent or pointed chin in infancy, prominent triangular jaw in adolescence		
Open‐mouthed expression		
Posteriorly rotated ears, large uplifted ear lobes with a central depression		
Seizures	79	+
Microcephaly^a^	78	+
Hypospadias in males	60	/
Congenital heart defects	58	+
Short stature^b^	46	+
Hirschsprung disease (HSCR)	44	‐
Cryptorchidism in males	41	/
Constipation (w/out known HSCR)	29	−
Renal anomalies	25	−
Structural eye anomalies	10	−
Pyloric stenosis	7	−
Pulmonary artery sling	3	−
Cleft palate	2	−
Thrombocytopenic purpura	Unreported	+

^a^ Head circumference ≥ 2 SD below the mean for age and sex.

^b^ Length or height ≥ 2 SD below the mean for age and sex.

Seizures are one of the most common neurologic issues in individuals with MWS, present in nearly 80% of patients.^4^, ^25^ The mean age of onset is approximately 3 years, and a first presentation of seizure as early as one month of age has been reported.^4^ The seizure occurred in our patient when she was 14 years old, which was later than the majority cases reported previously, and the mechanism was unclear. The patients with whole‐allele deletion are often associated with worsening features, particularly an earlier onset of seizures in Garavelli's study, but the numbers are small and can be used only for a descriptive analysis.^26^ More cases and further study need to be performed in the future.

The discovery of novel variants further expands the spectrum of known ZEB2 mutations associated with MWS in humans. In future studies, we will focus on developing transgenic animal models carrying the reported variants to characterize the mechanism by which ZEB2 deficiency leads to MWS.

## AUTHOR CONTRIBUTIONS

QP, CBR, SPL, BMZ, and XML conceived and designed the work; YH, QP, KZM, and CBR performed the experiments; YH, QP, SPL, KZM, and CBR analyzed the data; and YH, QP, and XML wrote the article. All authors read and approved the final article.

## ETHICS APPROVAL AND CONSENT TO PARTICIPATE

The study was approved by the Ethics Committee of Dongguan Children's Hospital in agreement with the Declaration of Helsinki. Written informed consent was obtained from the guardians of the study subject.

## CONSENT FOR PUBLICATION

Written informed consent was obtained from the guardian of the study subject.

## Data Availability

All relevant data are included in the article. The datasets used and/or analyzed during the current study are available from the corresponding author upon request.
